# Supplementary material for: REP-X: An Evolution-guided Strategy for the Rational Design of Cysteine-less Protein Variants
Source: Sci Rep. 2020 Feb 10;10:2193. doi: 10.1038/s41598-020-58794-x (PMC7010797; doi:10.1038/s41598-020-58794-x)
Supplement: Supplementary file 1 — Supporting Information. [file 41598_2020_58794_MOESM1_ESM.pdf]

REP-X: An Evolution-guided Strategy for the Rational Design of Cysteine-less Protein Variants

Kevin Dalton[1#], Tom Lopez[2], Vijay Pande[1,3]

and Judith Frydman[1,2]

[1]Biophysics Program, [2]Department of Biology, [3]Department of Chemistry

Stanford University, Stanford, California, USA

[#]Present address: Department of Molecular and Cellular Biology Harvard University, Cambridge, MA 02138

To whom correspondence should be addressed; E-mail:jfrydman@stanford.edu

Legends to Supplemental Figures

Figure S1: Distribution of Residues at Position 393 in Cpn orthologues

The observed distribution of amino acids at position 393 for protein sequences in the MmCpn homolog database. Distributions are shown for homologs with at least 70, 60, or 50 % sequence identity with MmCpn.

Figure S2: Non-denaturing agarose electrophoresis to monitor binding MmCpn and the substrate protein rhodanese. MmCpn migration in the open, unliganded state and the closed ATP-AlFx induced state are unaffected by the presence (left lanes) or absence (right lanes) of substrate protein rhodanese. Cy3-maleimide labeled rhodanese visualised by fluorescence imaging (top). Total protein stained with Sypro Ruby (bottom). Of note, Rhodanese alone does migrate into these gels due to its isoelectric point.

Table 1: ATPase rates of Mm-Cpn variants. Measurements of conversion of NADH to NAD+ was monitored by a decrease in absorbance at 340 nm for 120 seconds. The linear region of the timecourse was fit and the slope used to calculate the rate of ATP hydrolysis by MmCpn. ATPase rates are reported as mean +/- std error (n=3)

ATP Conc (μM)	MmCpn variant	ATPase rate
200.0	WT	0.816±0.014
	Cys-0	0.510±0.030
1000.0	WT	0.678±0.042
	Cys-0	0.450±0.052

Legends to Supplemental Datasets

Supplemental Data 1: Alignment between chaperonins of *Methanococcus maripaludis* and *Haloarcula hispanica* show 59.2% identity, yielding C359V

Supplemental Data 2: Alignment between chaperonins of *Methanococcus maripaludis* and *Thermococcus sp. 4557* show 66.3% identity, yielding C237E and C286V

Supplemental Data 3: Alignment between chaperonins of *Methanococcus maripaludis* and *Methanococcus thermolithotrophicus* show 85.8% identity, yielding C140M, C470Y and C484T

Supplemental Data 4: FASTA files of coding sequences used in this study

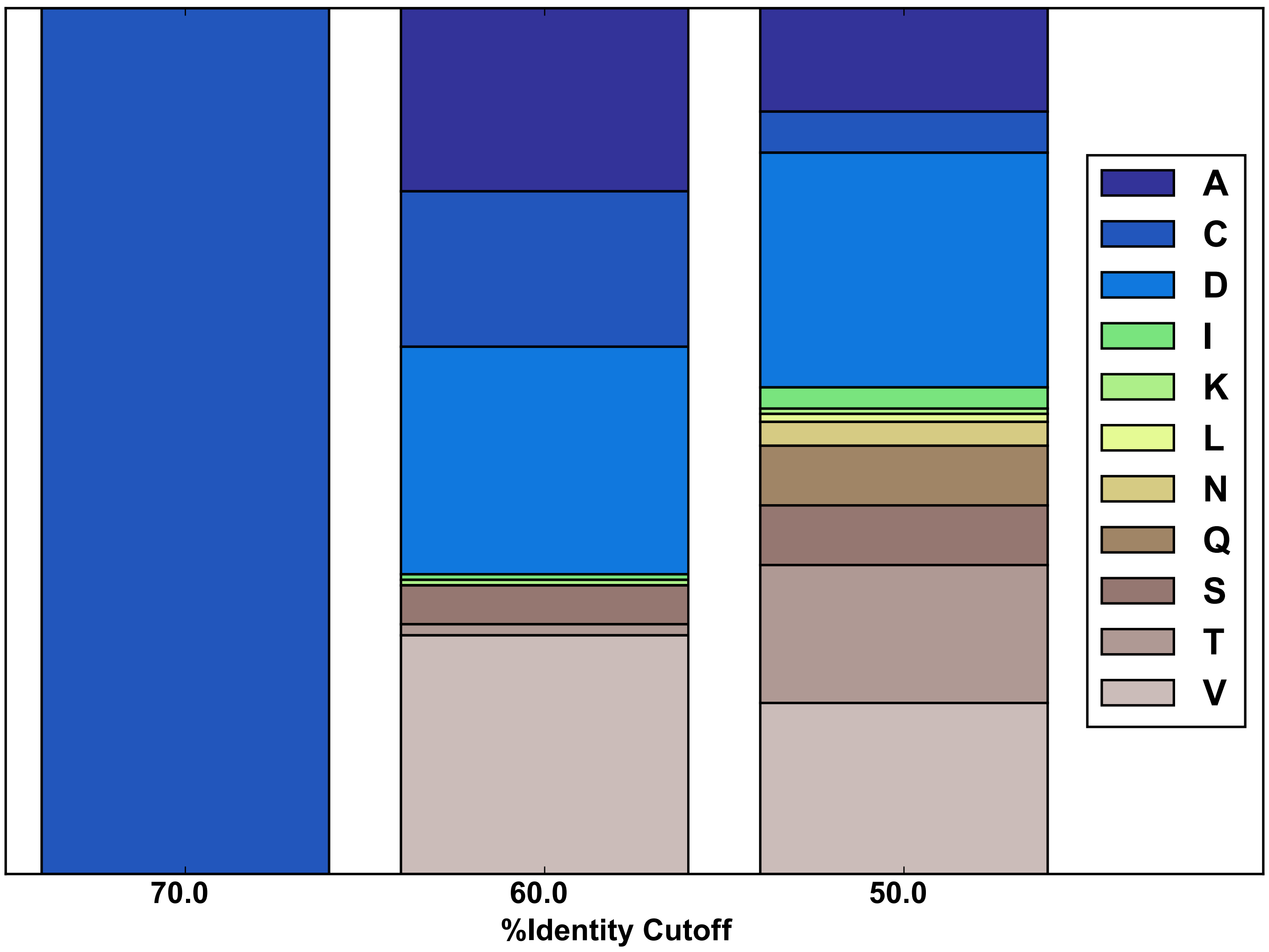
A
C
D
I
K
L
N
Q
S
T
V
70.0
60.0
50.0
%Identity Cutoff

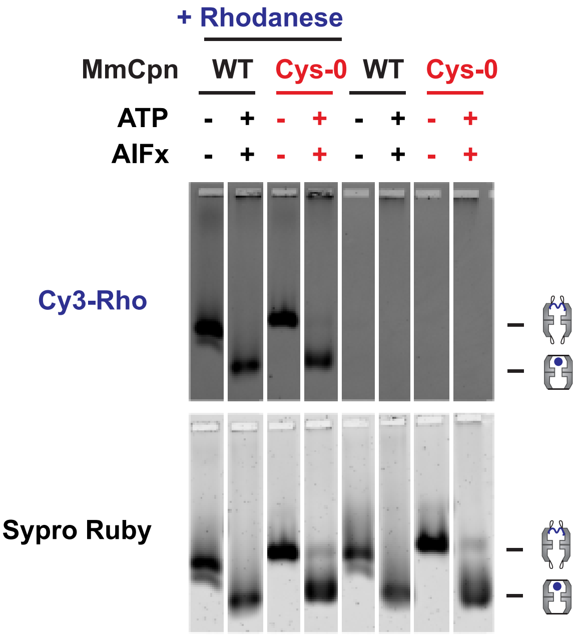
+ Rhodanese
MmCpn
WT
Cys-0
WT
Cys-0
ATP
- + - + - + - +
AlFx
- + - + - + - +
Cy3-Rho
Sypro Ruby

DALTON ET AL, SUPPLEMENTAL DATA 1

```
########################################
# Program: water
# Rundate: Wed 27 Nov 2019 11:06:13
# Commandline: water
#    [-asequence] maripaludis.fa
#    [-bsequence] haloarcula.fa
#    [-outfile] C359.txt
# Align_format: srspair
# Report_file: C359.txt
########################################

#=======================================
#
# Aligned_sequences: 2
# 1: AAM21720.1
# 2: AEM55790.1
# Matrix: EBLOSUM62
# Gap_penalty: 10.0
# Extend_penalty: 0.5
#
# Length: 539
# Identity:     319/539 (59.2%)
# Similarity:   422/539 (78.3%)
# Gaps:           3/539 ( 0.6%)
# Score: 1628.5
#
#
#=======================================

AAM21720.1         7
VLPENMKRYMGRDAQRMNILAGRIIAETVRSTLGPKGMDKMLVDDLGDVV     56

||.|..:|..|:|||.|||.||..:||.||:|||||||||||||:.|.||
AEM55790.1         3
VLSEESQRTSGKDAQSMNITAGTAVAEAVRTTLGPKGMDKMLVDNSGSVV     52

AAM21720.1        57
VTNDGVTILREMSVEHPAAKMLIEVAKTQEKEVGDGTTTAVVVAGELLRK    106

|||||||||.||.:|||||.|::|||:|||.|||||||||||:|||||.|
AEM55790.1        53
VTNDGVTILDEMDIEHPAANMIVEVAQTQEDEVGDGTTTAVVMAGELLSK    102

AAM21720.1       107
AEELLDQNVHPTIVVKGYQAAAQKAQELLKTIACEVGAQDKEILTKIAMT    156

|||||||::|.:|:.:||:.||:||:|:|:..|.:|.|.|.|.|.|:|.|
```

```
AEM55790.1       103
AEELLDQDIHASILAQGYRQAAEKAKEILEDNAIDVDADDTETLEKVAAT    152

AAM21720.1       157
SITGKGAEKAKEKLAEIIVEAVSAVVDDEGKVDKDLIKIEKKSGASIDDT    206
                     ::||||||.:|:.|||::|.|..:||||:|.||.|.|:||...|.:.|:
:
AEM55790.1       153
AMTGKGAESSKDILAELVVRAAQSVVDDDGSVDTDNIQIETVVGGATDES    202

AAM21720.1       207
ELIKGVLVDKERVSAQMPKKVTDAKIALLNCAIEIKETETDAEIRITDPA    256

||::||:||||||...||..|.||.:|||:.|||:.|||.|.|:.:|||.
AEM55790.1       203
ELVEGVIVDKERVHDNMPFAVEDADVALLDTAIEVPETELDTEVNVTDPD    252

AAM21720.1       257
KLMEFIEQEEKMLKDMVAEIKASGANVLFCQKGIDDLAQHYLAKEGIVAA    306
                     :|.:|::|||:.||:||.::|.:||:|:||||||||:||||||:|||:|
.
AEM55790.1       253
QLQQFLDQEEEQLKEMVDQLKEAGADVVFCQKGIDDMAQHYLAQEGILAV    302

AAM21720.1       307
RRVKKSDMEKLAKATGANVITNIKDLSAQDLGDAGLVEERKISGDSMIFV    356

||.||||:|.|:::|||.:|:||.|:.|.|||.||.|.::.|:||..|||
AEM55790.1       303
RRAKKSDIEALSRSTGARIISNIDDIEADDLGFAGSVAQKDIAGDERIFV    352

AAM21720.1       357
EECKHPKAVTMLIRGTTEHVIEEVARAVDDAVGVVGCTIEDGRIVSGGGS    406

|:.:..:||||::||.||||::||.||::|::|||..|:|||:::.|||:
AEM55790.1       353
EDVEDARAVTMILRGGTEHVVDEVERAIEDSLGVVAATLEDGKVLPGGGA    402

AAM21720.1       407
TEVELSMKLREYAEGISGREQLAVRAFADALEVIPRTLAENAGLDAIEIL    456
                     .|.:|::.||::|:.:.|||||||.|||||::||||||||||||||.|:.
|
AEM55790.1       403
PETQLALGLRDHADSVGGREQLAVEAFADAIDVIPRTLAENAGLDPIDSL    452

AAM21720.1       457
VKVRAAHASNGNKCAGLNVFTGAVEDMCENGVVEPLRVKTQAIQSAAEST    506

|.:|:.| ..|...:||:.:||.|.||.|:||||||||||||::||.|:.
AEM55790.1       453 VDLRSKH-
```

```
DGGAVTSGLDAYTGEVVDMEEDGVVEPLRVKTQAVESATEAA    501

AAM21720.1       507 EMLLRIDDVIAAEKLRG--APDMGDMGGMPGMGGMPGMM    543
                     .|:|||||||||..|:|  ..|..|.||..|.||.||.|
AEM55790.1       502 VMILRIDDVIAAGDLKGGQGDDDEDEGGPGGPGGAPGGM    540


#---------------------------------------
#---------------------------------------
```

DALTON ET AL, SUPPLEMENTAL DATA 2

```
########################################
# Program: water
# Rundate: Wed 27 Nov 2019 11:01:40
# Commandline: water
#    [-asequence] maripaludis.fa
#    [-bsequence] thermococcus.fa
#    [-outfile] C237-C286.txt
# Align_format: srspair
# Report_file: C237-C286.txt
########################################

#=======================================
#
# Aligned_sequences: 2
# 1: AAM21720.1
# 2: WP_014011858.1
# Matrix: EBLOSUM62
# Gap_penalty: 10.0
# Extend_penalty: 0.5
#
# Length: 546
# Identity:     362/546 (66.3%)
# Similarity:   450/546 (82.4%)
# Gaps:           7/546 ( 1.3%)
# Score: 1795.0
#
#
#=======================================

AAM21720.1         1 MSQQPGV-
LPENMKRYMGRDAQRMNILAGRIIAETVRSTLGPKGMDKMLV     49
                     :|.||.|
|||..:||:||||||:||||.||||||||:||||||||||||
WP_014011858.      4
LSGQPVVILPEGTQRYVGRDAQRLNILAARIIAETVRTTLGPKGMDKMLV     53

AAM21720.1        50
DDLGDVVVTNDGVTILREMSVEHPAAKMLIEVAKTQEKEVGDGTTTAVVV     99

|.||||||||||.|||..:.::||||||::||||||:||.|||||||||:
WP_014011858.     54
DSLGDVVVTNDGATILDRIDLQHPAAKMMVEVAKTQDKEAGDGTTTAVVI    103

AAM21720.1       100
AGELLRKAEELLDQNVHPTIVVKGYQAAAQKAQELLKTIACEVGAQDKEI    149

|||||||||||||||:||:|:||||..||:||||:|:.||.||...|:|.
WP_014011858.    104
```

```
AGELLRKAEELLDQNIHPSIIVKGYTMAAEKAQEILEDIAIEVTPDDEET    153

AAM21720.1       150 LTKIAMTSITGKGAEKAKEKLAEIIVEAVSAVVD-DEGK--
VDKDLIKIE    196
                     |.||||||||||.||..||..|::.|:||..|.: .:||
||.|.||||
WP_014011858.    154
LMKIAMTSITGKNAESHKELFAKLAVDAVRQVAEKKDGKFTVDIDNIKIE    203

AAM21720.1       197
KKSGASIDDTELIKGVLVDKERVSAQMPKKVTDAKIALLNCAIEIKETET    246

||:|.|::::||::||::|||||..:|||:|..|||||:|.|:|:|:|||
WP_014011858.    204
KKAGESVEESELVRGVVIDKERVHPRMPKRVEGAKIALINEALEVKKTET    253

AAM21720.1       247
DAEIRITDPAKLMEFIEQEEKMLKDMVAEIKASGANVLFCQKGIDDLAQH    296

||:|.||.|.:||.|:||||||||:||.:|.|:||||||.||||||||||
WP_014011858.    254
DAKINITSPDQLMSFLEQEEKMLKEMVDKIAATGANVLFVQKGIDDLAQH    303

AAM21720.1       297
YLAKEGIVAARRVKKSDMEKLAKATGANVITNIKDLSAQDLGDAGLVEER    346

||||.||:|.|||||||||||||||||.|:||:|||:|:|||.|.|||||
WP_014011858.    304
YLAKYGILAVRRVKKSDMEKLAKATGAKVVTNVKDLTAEDLGHADLVEER    353

AAM21720.1       347
KISGDSMIFVEECKHPKAVTMLIRGTTEHVIEEVARAVDDAVGVVGCTIE    396

||:|:||||||.||:|||||:||||.|||||:||.||::||:.||...:|
WP_014011858.    354
KIAGESMIFVEGCKNPKAVTILIRGGTEHVIDEVERALEDAIKVVKDVME    403

AAM21720.1       397
DGRIVSGGGSTEVELSMKLREYAEGISGREQLAVRAFADALEVIPRTLAE    446

||.::..||:.|:|||::|.|||:.:.|:|.||:..||:||::||:||||
WP_014011858.    404
DGYVLPAGGAPEIELSIRLDEYAKEVGGKEALAIENFAEALKIIPKTLAE    453

AAM21720.1       447
NAGLDAIEILVKVRAAHASNGNKCAGLNVFTGAVEDMCENGVVEPLRVKT    496
                     |||||.:|:||||.:.|.:.|
|..|::||.|...||..||::||||||.
WP_014011858.    454 NAGLDTVEMLVKVISEHKNKG-
KAIGIDVFEGEPADMLANGIIEPLRVKK    502
```

```
AAM21720.1       497 QAIQSAAESTEMLLRIDDVIAAEKLRGAPDMGDMGGMPGMGGMPGM
542
                     |||:||:|:..|:|||||||||:..:  |:.|..|||||||||.||
WP_014011858.    503 QAIKSASEAAIMILRIDDVIAAKVSK--PEGGQGGGMPGMGGMGGM
546


#---------------------------------------
#---------------------------------------
```

DALTON ET AL, SUPPLEMENTAL DATA 3

```
########################################
# Program: water
# Rundate: Wed 27 Nov 2019 11:09:41
# Commandline: water
#    [-asequence] maripaludis.fa
#    [-bsequence] methanothermococcus.fa
#    [-outfile] C140-C470-C484.txt
# Align_format: srspair
# Report_file: C140-C470-C484.txt
########################################

#=======================================
#
# Aligned_sequences: 2
# 1: AAM21720.1
# 2: BAA33889.1
# Matrix: EBLOSUM62
# Gap_penalty: 10.0
# Extend_penalty: 0.5
#
# Length: 535
# Identity:     459/535 (85.8%)
# Similarity:   497/535 (92.9%)
# Gaps:           2/535 ( 0.4%)
# Score: 2306.5
#
#
#=======================================

AAM21720.1         7
VLPENMKRYMGRDAQRMNILAGRIIAETVRSTLGPKGMDKMLVDDLGDVV     56

|||||:||:||||||||||||||||.||||||||||||||||||||||:|
BAA33889.1         9
VLPENVKRFMGRDAQRMNILAGRIIGETVRSTLGPKGMDKMLVDDLGDIV     58

AAM21720.1        57
VTNDGVTILREMSVEHPAAKMLIEVAKTQEKEVGDGTTTAVVVAGELLRK    106

|||||||||:||||||||||||||||||||||||||||||||:|||||||
BAA33889.1        59
VTNDGVTILKEMSVEHPAAKMLIEVAKTQEKEVGDGTTTAVVIAGELLRK    108

AAM21720.1       107
AEELLDQNVHPTIVVKGYQAAAQKAQELLKTIACEVGAQDKEILTKIAMT    156

||||||||||||||:||||.|.|||||:||.||.:|.|.|||||.|||||
BAA33889.1       109
```

```
AEELLDQNVHPTIVIKGYQLAVQKAQEVLKEIAMDVKADDKEILHKIAMT    158

AAM21720.1       157
SITGKGAEKAKEKLAEIIVEAVSAVVDDEGKVDKDLIKIEKKSGASIDDT    206

||||||||||||||.|:|||||:||||:.|||||||||||||.|||:|:|
BAA33889.1       159
SITGKGAEKAKEKLGEMIVEAVTAVVDESGKVDKDLIKIEKKEGASVDET    208

AAM21720.1       207
ELIKGVLVDKERVSAQMPKKVTDAKIALLNCAIEIKETETDAEIRITDPA    256

|||.|||:||||||.|||||:.:||||||||.||:||||||||||||||.
BAA33889.1       209
ELINGVLIDKERVSPQMPKKIENAKIALLNCPIEVKETETDAEIRITDPT    258

AAM21720.1       257
KLMEFIEQEEKMLKDMVAEIKASGANVLFCQKGIDDLAQHYLAKEGIVAA    306

|||||||||||||||||..||||||||||||||||||||||||||||:|.
BAA33889.1       259
KLMEFIEQEEKMLKDMVDTIKASGANVLFCQKGIDDLAQHYLAKEGILAV    308

AAM21720.1       307
RRVKKSDMEKLAKATGANVITNIKDLSAQDLGDAGLVEERKISGDSMIFV    356

|||||||||||:|||||||:||||||.|:|||:||:||||||:||:||||
BAA33889.1       309
RRVKKSDMEKLSKATGANVVTNIKDLKAEDLGEAGIVEERKIAGDAMIFV    358

AAM21720.1       357
EECKHPKAVTMLIRGTTEHVIEEVARAVDDAVGVVGCTIEDGRIVSGGGS    406

|||||||||||||||||||||||||||||||:|||.||||||:||:|||:
BAA33889.1       359
EECKHPKAVTMLIRGTTEHVIEEVARAVDDAIGVVACTIEDGKIVAGGGA    408

AAM21720.1       407
TEVELSMKLREYAEGISGREQLAVRAFADALEVIPRTLAENAGLDAIEIL    456
                     .|:||:||||:||||:|||||||||||||||||:||||||||||||||:
|
BAA33889.1       409
AEIELAMKLRDYAEGVSGREQLAVRAFADALEVVPRTLAENAGLDAIEML    458

AAM21720.1       457
VKVRAAHASNGNKCAGLNVFTGAVEDMCENGVVEPLRVKTQAIQSAAEST    506

||:||.||...|...|||||||.||:|.||||||||||||||||||.|:|
BAA33889.1       459
VKLRAKHAEGNNAYYGLNVFTGDVENMTENGVVEPLRVKTQAIQSATEAT    508
```

```
AAM21720.1       507 EMLLRIDDVIAAEKLRGAP--DMGDMGGMPGMGGM    539
                     |||||||||||||||.|..  ||||||||.|||||
BAA33889.1       509 EMLLRIDDVIAAEKLSGGSGGDMGDMGGMGGMGGM    543
```

```
#---------------------------------------
#---------------------------------------
```

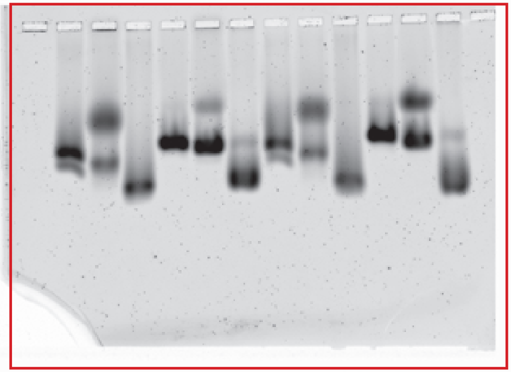

Fig 3 and Fig S2 Sypro Ruby staining

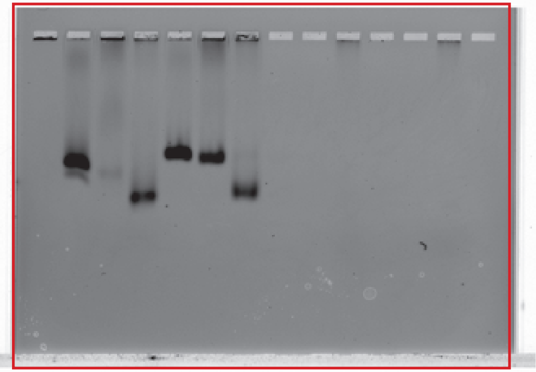

Fig 3 and Fig S2 Cy3-Rhodanese

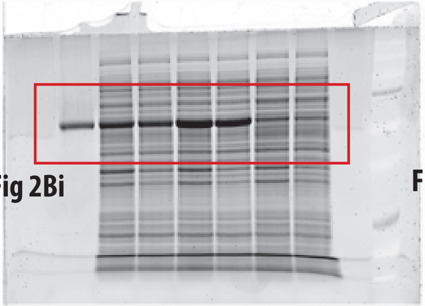

Fig 2Bi

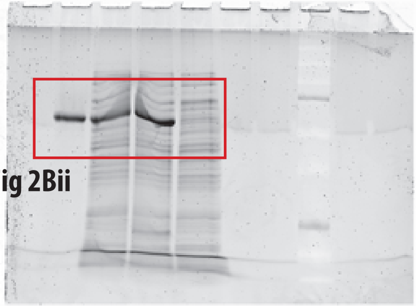

Fig 2Bii

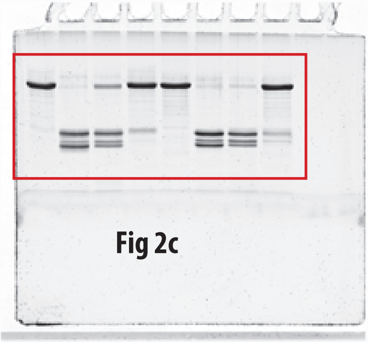

Fig 2c

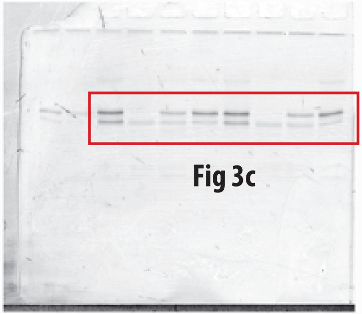

Fig 3c
